# Predicting the ideal apex of lumbar lordosis based on individual pelvic incidence and inflection point in asymptomatic adults

**DOI:** 10.3389/fsurg.2022.912357

**Published:** 2022-09-29

**Authors:** Jingyu Wang, Qianshi Zhang, Fubing Liu, Hui Yuan, Yi Zhang, Xiaobin Wang, Jing Li

**Affiliations:** Department of Spine Surgery, Spinal Deformity Center, The Second Xiangya Hospital of Central South University, Changsha, China

**Keywords:** apex of lumbar lordosis, inflection point, lumbar lordosis, pelvic incidence, sagittal alignment

## Abstract

**Objective:**

The main aim of this study was to comprehensively explore the relationship among pelvic incidence (PI), inflection point (IP), and apex of lumbar lordosis (LLA), and establish a predictive formula for LLA based on individual PI and IP in asymptomatic Chinese adults.

**Methods:**

A total of 385 asymptomatic adults with average age 38.3 ± 11.9 years (range 20–73 years) were recruited between November 2020 and October 2021. Full-spine, standing x-rays were then obtained from each participant. Next, the following sagittal parameters were measured: PI, IP, LLA, the horizontal offset between the plumb line of the lumbar apex and that of the posterosuperior corner of S1 (LASO), the upper lumbar lordosis (ULL) and lower lumbar lordosis (LLL), lumbar lordosis (LL), and thoracic kyphosis (TK). Moreover, the association among PI, IP, and the other sagittal parameters was evaluated, followed by linear regression analyses. A *P*-value of <0.05 was considered statistically significant.

**Results:**

PI showed statistically significant correlations with LLA (*r_s_* = −0.629; *P* < 0.01), LASO (*r_s_* = 0.537; *P* < 0.01), LLL (*r_s_* = 0.788; *P* < 0.01), and LL (*r_s_* = 0.663; *P* < 0.01). On the other hand, IP also showed statistically significant correlations with LLA (*r_s_* = 0.671; *P* < 0.01), LASO (*r_s_* = −0.493; *P* < 0.01), LLL (*r_s_* = −0.402; *P* < 0.01), and LL (*r_s_* = −0.283; *P* < 0.01). The corresponding predictive formulae were displayed as follows: LLA = −0.03 * PI + 0.23 * IP + 14.45 (*R*^2 ^= 0.669); LASO = 0.38 * PI−2.09 * IP + 53.62 (*R*^2 ^= 0.460); and LLL = 0.58 * PI−0.88 * IP + 18.86 (*R*^2 ^= 0.659).

**Conclusion:**

The specific lumbar shape should be modulated by pelvic morphology and IP level. In addition, we established predictive formulae for ideal sagittal lumbar profile based on individual PI and IP, with the overarching goal of helping surgeons to better comprehend the regulatory mechanisms of the individual sagittal lumbar alignment, and design a precise and personalized corrective plan.

## Introduction

In recent years, sagittal balance in spinal curvature has increasingly become a research hotspot. Sagittal imbalance in patients with lumbar degenerative diseases is significantly associated with poor health-related quality of life (1, 2). Moreover, studies have revealed that inappropriate sagittal alignment significantly increases the incidence of mechanical complications after correction surgery (3–6). Therefore, achieving physiological sagittal alignment, especially the normal lumbar lordosis, is of prime importance.

Roussouly et al. (7) proposed four types of lumbar lordosis in asymptomatic adults based on the sacral slope (SS) orientation, with each type possessing a distinct sagittal spinopelvic morphology. However, SS is a positional parameter, influenced by pelvic rotation, and cannot be used as a suitable tool to classify sagittal morphology in pathological states; pelvic incidence (PI), a morphological parameter independent of positioning of the pelvis, is closely correlated with sagittal lumbar parameters (8, 9); therefore, SS was gradually replaced by PI to describe the sagittal types (3, 10). Previous studies found that restoring the ideal Roussouly sagittal profile according to individual PI can significantly decrease the risk of mechanical complications in adult spinal deformity (ASD) surgery (3, 4). The apex of lumbar lordosis (LLA), which divides the lordosis into upper and lower arcs, can affect the sagittal lumbar profile. Besides, ideal LLA positioning plays a vital role in restoring a reasonable lumbar profile for ASD patients (11). Sebaaly et al. (12) suggested that the optimal LLA should be L4 when PI < 55° and L3 when PI ≥ 55°; although the principle is simple and useful, it is not quantitative. Additionally, some scholars proposed restoring the ideal LLA based on individualized PI, e.g., LLA = −0.042*PI + 6.134 (13); however, the impact of inflection point (IP) on LLA has been ignored. For example, type 1 and type 2, with similar PI but different IP, results in two diverse kinds of sagittal lumbar profiles.

The main aim of this study was to comprehensively explore the relationship among PI, IP, and LLA, and establish a predictive formula for LLA based on individual PI and IP in asymptomatic Chinese adults. By virtue of the predictive model, we aimed at providing the reference values of sagittal lumbar parameters to customize a more precise surgical correction strategy for ASD patients.

## Materials and methods

### Patient population

A total of 412 asymptomatic Chinese adults [including 218 males and 194 females, with a mean age of 37.5 ± 11.1 years (ranging from 20 to 73 years old)] were recruited from Hunan Province between November 2020 and October 2021. The following inclusion criteria were used: (1) no spinal pathology or deformity; (2) no history of pelvic, hip, or lower limbs disease; (3) no history of spinal surgery; and (4) no neurological or neuromuscular disorder.

**Figure 1:**
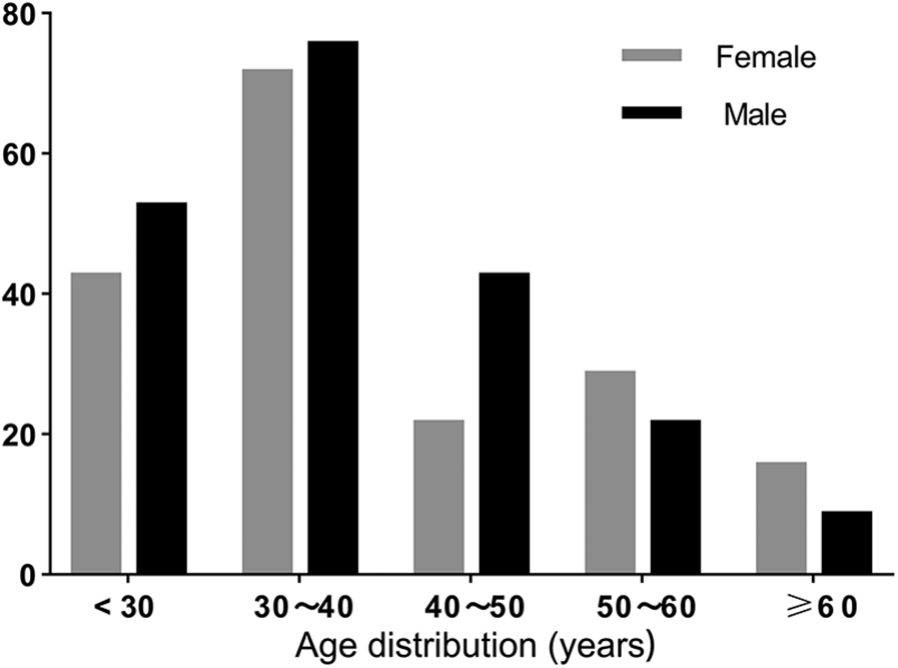
The composition of participants in each age stratification.

Among the enrolled population, 27 subjects were excluded [10 with ambiguous anatomical structure in the films, eight with a sagittal vertical axis (SVA) larger than 50 mm, four with coronal scoliosis of more than 10°, and five with lumbar spondylolisthesis]. Consequently, a total of 385 subjects, including 203 males and 182 females, were eventually included in the derivation cohort to develop a novel predictive formula for LLA. The mean age of the subjects was 38.3 ± 11.9 years (range 20–73 years). The composition of participants in each age stratification was as follows: 96 (24.9%, 53 men and 43 women) aged <30 years, 148 (38.4%, 76 men and 72 women) in their 30s, 65 (16.9%, 43 men and 22 women) in their 40s, 51 (13.2%, 22 men and 29 women) in their 50s, and 25 (6.5%, 9 men and 16 women) aged ≥60 years (Figure 1).

Besides, a cohort of 50 asymptomatic Chinese adults were recruited from November 2021 to January 2022 for validation of the newly established formula.

It is worth noting that this study was approved by the ethics committee of our institution, and signed informed consent was obtained from all participants prior to the study.

### Radiographic measurements

Whole-spine x-rays were obtained from each participant in an erectly standing posture at a 90° position (the arms and elbows straight out with hands gently grasping a pole) or clavicle position (the elbows fully bent with hands placed into the supraclavicular fossae) (14).

**Figure 2:**
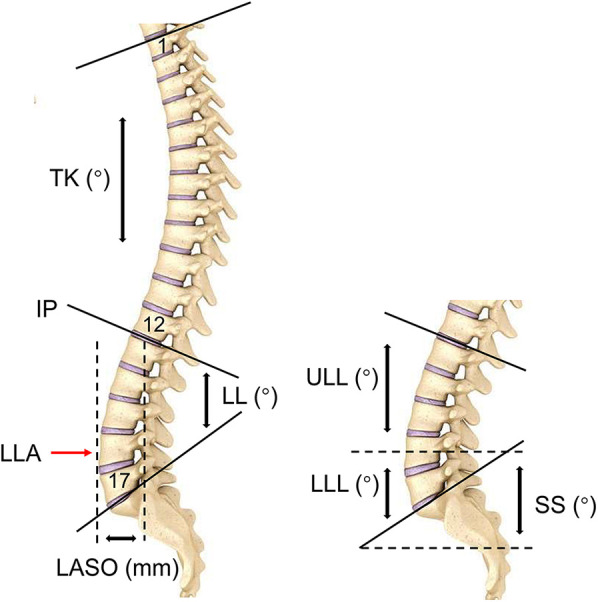
The descriptions of sagittal parameters. IP, inflection point; LLA, apex of lumbar lordosis; LASO, the horizontal offset between the plumb line of the lumbar apex and that of the posterosuperior corner of S1; ULL, upper lumbar lordosis; LLL, lower upper lumbar lordosis; LL, lumbar lordosis; TK, thoracic kyphosis.

Vertebrae from T1 to L5 were coded with numbers 1–17 for data collection and statistical analysis. When the point was located between two adjacent vertebrae, it was coded as the number of superior vertebra plus 0.5 (15). Notably, IP corresponded to the most inclined vertebrae or disc at the transition from lordosis to kyphosis (7), whereas LLA was defined as the most anterior vertebra or disc touched by the plumb line (7). Moreover, we measured the horizontal offset between the plumb line of the lumbar apex and that of the posterosuperior corner of S1 (LASO) (16). LL was defined as the Cobb angle bound by the IP and the superior endplate of S1. LL was divided by the horizontal line passing through the LLA into two arcs, the upper lumbar lordosis (ULL) and lower lumbar lordosis (LLL). Thoracic kyphosis (TK) indicated the Cobb angle formed between the superior endplate of T1 and the IP. In addition, PI was the angle subtended by a perpendicular line from the midpoint of the S1 endplate and a line connecting this point to the center of femoral heads. The SS was geometrically equal to the LLL (Figure 2).

All radiographic parameters were measured by two experienced spinal surgeons, and the average of two measurements was used in data analysis. Intra- and inter-observer reliability was evaluated in 50 randomly selected participants using intraclass correlation coefficient (ICC). The intra-observer ICCs for PI, IP, LLA, LASO, LLL, LL,TK were 0.952, 0.904, 0.913, 0.921, 0.930, 0.962 and 0.901, while inter-observer ICCs for the aforementioned variables were 0.915, 0.897, 0.906, 0.927, 0.942, 0.935 and 0.920, respectively. Based on the Shrout and Fleiss criteria (17), both intra- and inter-observer reliability were satisfactory.

### Statistical analyses

All statistical analyses were performed using SPSS 23.0 (SPSS Inc., Chicago, Illinois, USA) software. All data are expressed as mean ± standard deviation. The Kolmogorov-Smirnov/Shapiro-Wilk test was used to determine the normality of variables. Correlations between the sagittal parameters were analyzed using the Spearman or Pearson correlation coefficient, followed by linear regression analyses. Correlation strengths were assessed and characterized as very weak (*r_s_* = 0.00–0.190), weak (*r_s_* = 0.20–0.39), moderate (*r_s_* = 0.40–0.59), strong (*r_s_* = 0.60–0.79), or very strong (*r_s_* = 0.80–1.00) (18). Independent *t*-test/Paired *t*-test was used to compare the differences between two groups, and analysis of variance between multiple groups was performed using one-way ANOVA. For the data that did not follow a normal distribution, Mann-Whitney test/Wilcoxon Signed Ranks test and Kruskal-Wallis test were used to analysis the differences between two and multiple groups, respectively. A *P*-value of <0.05 was considered statistically significant.

## Results

In this study, none of the variables followed a normal distribution except LL, ULL and LLL(SS), and wide fluctuation was observed in all sagittal parameters. PI averaged 46.8 ± 9.7°, ranging from 22.8° to 82.6°; IP averaged 12.7 ± 1.2 (approximately at L1), ranging from 7 (T7) to 15 (L3); the LLA was 16.1 ± 0.5 (approximately at L4) on average, ranging from 14.0 (L2) to 17.5 (L5/S1); and the mean value of the LASO was 44.9 ± 7.3 mm, ranging from 28.2 to 77.1 mm. Table 1 shows the characteristics of the specific sagittal parameters.

**Table 1:** Description of demographic and sagittal spinopelvic parameters.

Variables	Mean	Minimum	Maximum	Standard deviation
Age (years)	38.3	20	73	11.9
Female/male (*n*)	182/203			
PI (°)	46.8	22.8	82.6	9.7
IP	12.7	7.0	15.0	1.2
LL (°)	55.9	33.2	90.3	9.1
LLA	16.1	14.0	17.5	0.5
LASO (mm)	44.9	28.2	77.1	7.3
ULL (°)	20.9	7.1	35.9	4.8
LLL or SS (°)	35.0	14.4	58.3	7.4
TK (°)	40.3	13.7	69.8	8.7

PI, pelvic incidence; IP, inflection point; LL, lumbar lordosis; LLA, apex of lumbar lordosis; LASO, the horizontal offset between the plumb line of the lumbar apex and that of the posterosuperior corner of S1; ULL, upper lumbar lordosis; LLL, lower lumbar lordosis; SS, sacral slope; TK, thoracic kyphosis.

First, participants were stratified according to age and gender. No statistical differences in IP across age groups or genders were observed. In addition, no significant difference in PI was found between age groups, while the PI for female appeared to be slightly higher than that for male, although the difference was not statistically significant in this study (Table 2). Second, stratification of IP based on PI and gender was performed. Interestingly, IP tends to ascend slightly as PI increases for females (*P* < 0.001), while this trend is less pronounced among males (*P* = 0.047) (Table 3).

**Table 2:** The age stratified PI and IP in asymptomatic Chinese adults with different gender groups.

Age (years)	Age < 30	30 ≤ Age < 40	40 ≤ Age < 50	50 ≤ Age < 60	60 ≤ Age	*P*1
F/M (*n*)	43/53	72/76	22/43	29/22	16/9	
PI (°)						
Female	45.1 ± 10.6	47.5 ± 11.5	46.4 ± 9.0	49.6 ± 8.4	50.4 ± 11.1	0.300
Male	44.6 ± 8.8	46.6 ± 9.6	45.8 ± 7.8	48.7 ± 7.3	49.6 ± 8.9	0.123
*P*2	0.774	0.095	0.508	0.350	0.692	
IP						
Female	13.2 ± 1.1	12.8 ± 1.1	12.8 ± 1.2	12.5 ± 1.3	13.1 ± 0.8	0.330
Male	12.2 ± 1.7	12.6 ± 1.3	12.9 ± 0.8	12.6 ± 1.2	13.1 ± 0.6	0.193
*P*3	0.050	0.471	0.547	0.942	0.934	

F, female; M, male; PI, pelvic incidence; IP, inflection point. *P*1, differences in PI or IP between age subgroups; *P*2, differences in PI between genders in each age subgroup; *P*3, differences in IP between genders in each age subgroup.

**Table 3:** The PI stratified IP in asymptomatic Chinese adults with different gender groups.

PI (°)	PI < 35	35 ≤ PI < 45	45 ≤ PI < 55	55 ≤ PI	*P*1
Female/male (*n*)	22/17	60/79	57/78	43/29	
IP (mean, mode)					
Female	13.7 ± 0.9 (14)	13.1 ± 1.2 (13)	12.6 ± 1.1 (13)	12.5 ± 0.7 (12)	<0.001
Male	12.8 ± 1.8 (13)	12.6 ± 1.4 (13)	12.6 ± 1.2 (13)	12.2 ± 1.2 (12)	0.047
*P*2	0.098	0.236	0.692	0.385	

PI, pelvic incidence; IP, inflection point. *P*1, differences in IP between PI subgroups; *P*2, differences in IP between genders in each PI subgroup.

Results showed that PI and IP had statistically significant correlations with LLA, LASO, LLL or SS, and LL (Table 4). In addition, no significant relationship was found between LLL and ULL, however, the correlation between LLL and LL (*r_s_*_ _= 0.854) is stronger than that between ULL and LL (*r_s_*_ _= 0.594). TK was significantly correlated with ULL (*r_s_* = 0.629, *P* < 0.01, TK = 1.20*ULL + 15.23, *R*^2 ^= 0.426), but not with PI and IP.

**Table 4:** Correlation coefficient of the spinopelvic parameters.

Parameters	PI (°)	IP	LLA	LASO (mm)	ULL (°)	LLL or SS (°)	LL (°)	TK (°)
PI (°)		−0.318*	−0.629*	0.537*	0.057	0.788*	0.663*	0.051
IP			0.671*	−0.493*	0.069	−0.402*	−0.283*	0.004
LLA				−0.696*	0.184*	−0.639*	−0.408*	0.144*
LASO (mm)					−0.096	0.541*	0.379*	−0.023
ULL (°)						0.089	0.594*	0.629*
LLL or SS (°)							0.854*	0.044
LL (°)								0.370*

PI, pelvic incidence; IP, inflection point; LL, lumbar lordosis; LLA, apex of lumbar lordosis; LASO, the horizontal offset between the plumb line of the lumbar apex and that of the posterosuperior corner of S1; ULL, upper lumbar lordosis; LLL, lower lumbar lordosis; SS, sacral slope; TK, thoracic kyphosis.

* *P* < 0.01.

Table 5 shows the results of multiple linear regressions. According to the results, PI and IP were two significant predictors in the LLA, LASO, and LLL(SS) models. The corresponding predictive formulae are displayed as follows:

**Table 5:** Stepwise multiple linear regressions.

Dependent variables	Independent variables	*β*	Standard error	*t*	*P*	*R* ^2^
LLA	Constant	14.45	0.20	71.81	<0.001	0.669
	PI (°)	−0.03	0.00	−16.84	<0.001	
	IP	0.23	0.01	17.74	<0.001	
LASO (mm)	Constant	53.62	3.48	15.43	<0.001	0.460
	PI (°)	0.38	0.03	13.09	<0.001	
	IP	−2.09	0.23	−9.14	<0.001	
LLL or SS (°)	Constant	18.86	2.80	6.74	<0.001	0.659
	PI (°)	0.58	0.02	24.97	<0.001	
	IP	−0.88	0.18	−4.78	<0.001	

PI, pelvic incidence; IP, inflection point; LLA, apex of lumbar lordosis; LASO, the horizontal offset between the plumb line of the lumbar apex and that of the posterosuperior corner of S1; LLL, lower lumbar lordosis; SS, sacral slope.


$$\mathrm{LLA}=-0.03*\mathrm{PI}+0.23*\mathrm{IP}+14.45\left(R^{2}=0.669\right)$$

$$\mathrm{LASO}=0.38*\mathrm{PI}-2.09*\mathrm{IP}+53.62\left(R^{2}=0.460\right)$$

$$\mathrm{LLL}\;\mathrm{or}\;\mathrm{SS}=0.58*\mathrm{PI}-0.88*\mathrm{IP}+18.86\left(R^{2}=0.659\right)$$

Among 50 subjects in the validation cohort, 21 were males and 29 were females with an average age of 37.9 ± 12.8 years. Subjects in the derivation cohort and the validation cohort were matched in terms of gender, age, PI and IP (Table 6). Then, the theoretical LLA, LASO and LLL(SS) of participants in the validation cohort were determined using PI and IP based on the established formulae. Results of the comparison of actual LLA, LASO and LLL(SS) with their predicted values showed no significant differences (Table 7).

**Table 6:** Baseline characteristics of the 2 cohorts.

Variables	Derivation cohort	Validation cohort	*P* value
Age (years)	38.3 ± 11.9	37.9 ± 12.8	0.811
Gender (male/female)	203/182	21/29	0.153
PI (°)	46.8 ± 9.7	46.6 ± 8.8	0.731
IP	12.7 ± 1.2	12.8 ± 0.9	0.954

PI, pelvic incidence; IP, inflection point.

**Table 7:** Comparison between actual and predicted values of spinopelvic parameters in the validation cohort.

Variables	Actual value	Predicted value	*P* value
LLA	16.1 ± 0.5	16.0 ± 0.4	0.103
LASO (mm)	43.6 ± 5.0	44.6 ± 4.2	0.233
LLL or SS (°)	33.9 ± 7.2	34.6 ± 5.4	0.304

LLA, apex of lumbar lordosis; LASO, the horizontal offset between the plumb line of the lumbar apex and that of the posterosuperior corner of S1; LLL, lower lumbar lordosis.

## Discussion

Restoring the ideal lumbar lordosis not only refers to a proper magnitude, but also a reasonable shape. Given that the position of LLA affects the lumbar spine morphology, correct restoration of its location based on Roussouly classification helps to decrease the occurrence of mechanical complications after ASD surgery. For instance, Sebaaly et al. (12) found a higher incidence of PJK in cases where LLA did not match the ideal Roussouly classification (OR = 4.6), and a deviation of the LLA from the ideal position by two segments was associated with an even higher PJK incidence (as high as 75%); Pizones et al. (11) analyzed the influence of mismatch between LLA and ideal location on mechanical complication, they found that the incidence of mechanical complications was 66.7% when LLA was 0.5 segment lower than the ideal location, and 100% when the LLA was higher than the ideal position by three segments. However, Roussouly classification offers only qualitative descriptions of lumbar profiles, instead of individualized values of LLA and magnitude of LL needed for patients with ASD.

Previous studies have demonstrated that there is a correlation between PI, an individual anatomical parameter that reflects the pelvic morphology, and the sagittal lumbar parameters (8, 9). Notably, the IP represents the position where lordosis curvature turns into kyphosis, and the location of the IP can determine the length of actual lordosis. With regard to the relationships between LLA and sagittal spinopelvic parameters, only a handful of studies have explored the simple linear correlations of LLA with PI and IP. For example, Roussouly et al. (7) found that the LLA was statistically correlated with IP (*r_s_*_ _= 0.52, *P* < 0.01) in 160 asymptomatic Caucasian volunteers, whereas Pan et al. (13) reported that there were statistical correlations between PI and LLA (*r_s_* = −0.595, *P* < 0.01) in 183 asymptomatic Chinese adults. To date, there has been an absence of relevant studies that have comprehensively explored LLA by incorporating the impacts of PI and IP. Therefore, studies should be conducted with the aim of helping surgeons better comprehend the regulatory mechanisms of the individual sagittal lumbar profile and reconstruct an ideal LLA for ASD patients.

In the current study, we described the position of the lumbar apex in two dimensions; the longitudinal vertebra level (LLA) and its horizontal offset to the plumb line of the posterosuperior corner of S1 (LASO) (19). A significant correlation was found between PI and LLA (*r_s_* = −0.629, *P* < 0.01), and between PI and LASO (*r_s_* = 0.537, *P* < 0.01), indicating that the location of the LLA is determined by the pelvis morphology. This suggests that as the PI increases, the LLA moves more cranially and the lumbar curvature becomes prominent. On the other hand, LLA (*r_s_* = 0.671, *P* < 0.01) and LASO (*r_s_* = −0.493, *P* < 0.01) were statistically correlated with IP, suggesting that the location of LLA is also restricted by the IP level. Therefore, as the IP level descends, the LLA moves more caudally and the lordosis becomes flat.

**Figure 3:**
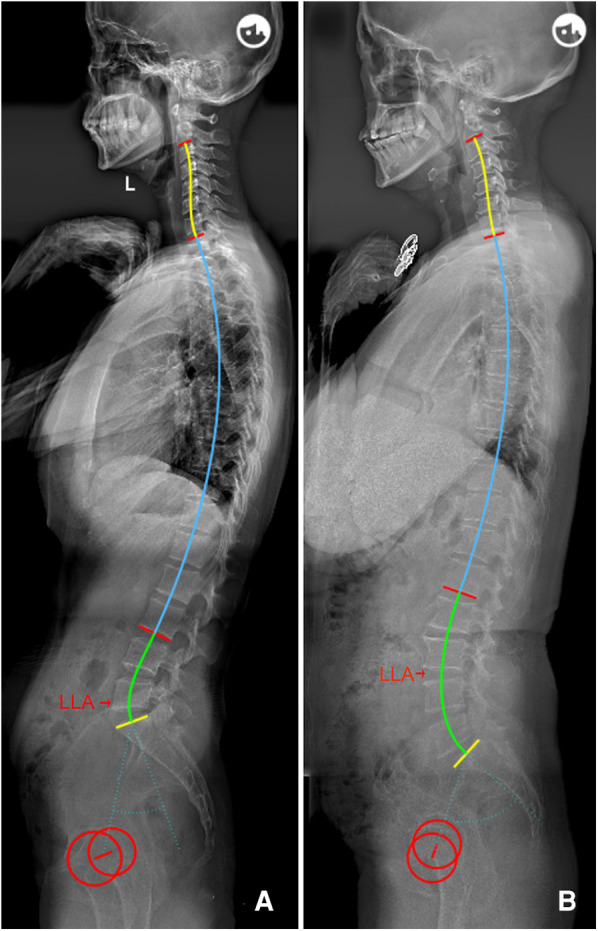
**(A**) Female, 29 years old, with a low PI (32.4°) and low IP (15); LLA = 17, LASO = 32.5 mm, LLL = 20.5°, ULL = 25.1°, and LL = 45.6°. (**B**) Female, 64 years old, with a higher PI (65.8°) and IP (13); LLA = 15.5, LASO = 51.6 mm, LLL = 47.7°, ULL = 19.7°, and LL = 67.4°. PI, pelvic incidence; IP, inflection point; LLA, apex of lumbar lordosis; LASO, the horizontal offset between the plumb line of the lumbar apex and that of the posterosuperior corner of S1; LLL, lower lumbar lordosis; ULL, upper lumbar lordosis; LL, lumbar lordosis.

**Figure 4:**
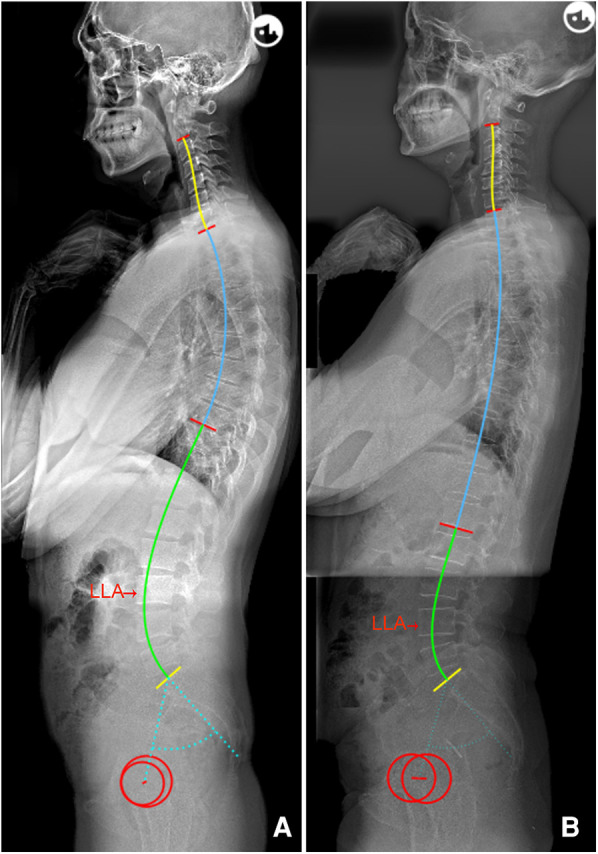
(**A**) Male, 29 years old, PI = 54.4°, IP = 9; LLA = 15, LASO = 50.7 mm, LLL = 42.2°, ULL = 24.8°, and LL = 67.0°. (**B**) Female, 51 years old, with a similar PI (55.0°) to a, but a lower IP (13); LLA = 16, LASO = 46.7 mm, LLL = 39.5°, ULL = 15.7°, and LL = 55.2°. PI, pelvic incidence; IP, inflection point; LLA, apex of lumbar lordosis; LASO, the horizontal offset between the plumb line of the lumbar apex and that of the posterosuperior corner of S1; LLL, lower lumbar lordosis; ULL, upper lumbar lordosis; LL, lumbar lordosis.

**Figure 5:**
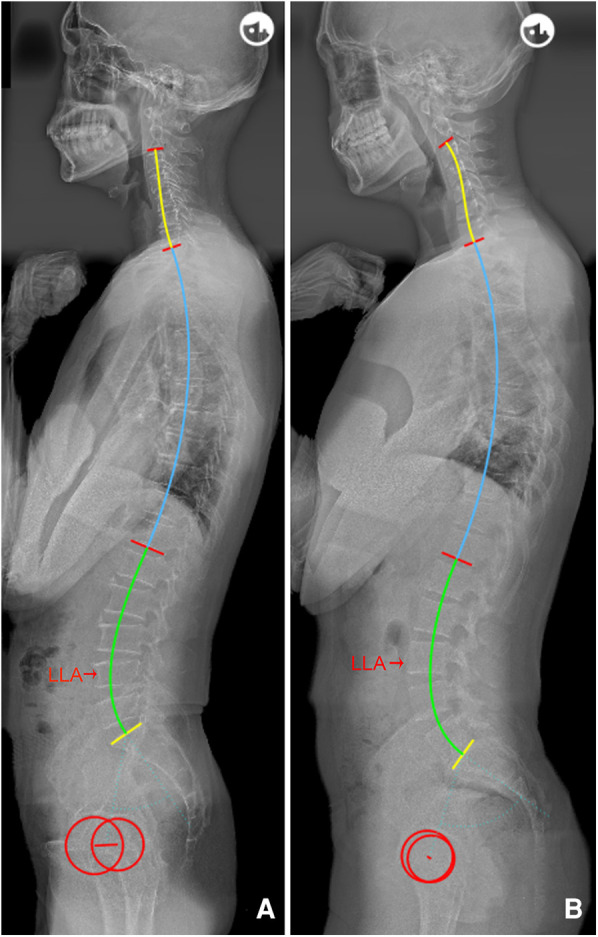
(**A**) Male, 55 years old, PI = 44.8°, IP = 12; LLA = 16, LASO = 46.7 mm, LLL = 35.0°, ULL = 25.2°, and LL = 60.2°. (**B**) Male, 23 years old, with the same IP (12) as a, but with a higher PI (72.2°); LLA = 15, LASO = 60.2 mm, LLL = 53.2°, ULL = 25.3°, and LL = 78.5°. PI, pelvic incidence; IP, inflection point; LLA, apex of lumbar lordosis; LASO, the horizontal offset between the plumb line of the lumbar apex and that of the posterosuperior corner of S1; LLL, lower lumbar lordosis; ULL, upper lumbar lordosis; LL, lumbar lordosis.

The following novel formulae were developed through multiple linear regressions: LLA = −0.03 * PI + 0.23 * IP + 14.45 (*R*^2 ^= 0.669) and LASO = 0.38 * PI − 2.09 * IP + 53.62 (*R*^2 ^= 0.460). The above models intuitively suggest that the lumbar apex is determined by individual PI and IP. In other words, decreased PI value and IP level should be accompanied by a short and flat lordosis with the LLA located caudally, corresponding to Roussouly type 1. Conversely, a large PI and high IP level suggests that the LLA should be located more superiorly and away from the line of gravity, thereby resulting in a long and ample lumbar curve corresponding to type 4 (Figure 3). In addition, even individuals with similar PI will present different location of LLA due to variation in IP (Figure 4). Notably, our findings are also supported by the Roussouly classification. For example, a low PI combined with different IP levels constitutes two kinds of lumbar shapes, type 1 and type 2. Similarly, the location of LLA and degree of lumbar curve are different for individuals with different PI values, even in instances where the IP level is the same (Figure 5). Therefore, the varying lumbar shape can be attributed to the diversity of pelvic morphology and IP level.

The prediction efficiency of the formula (*R*^2^) was 0.669, indicating that 66.9% of the total variation observed in LLA can be explained by PI and IP. Thus, the developed formulae outperformed formulae containing a single variable, such as LLA = −0.042 * PI + 6.134 (*R*^2 ^= 0.306) (13) and LLA = 0.188 * IP + 13.798 (*R*^2 ^= 0.299) (15). Moreover, results from our model (LLA = −0.03 * PI + 0.23 * IP + 14.45) showed a positive correlation between LLA and IP (*β* = 0.23, *P* < 0.001), suggesting that the choice of IP level will largely determine the location of LLA since PI is relatively constant for a specific individual. Namely, for every two levels that the IP descends, the LLA should be positioned more caudally by approximate 0.5 segments, and vice versa. Hey et al. (20) explored the influence of IP on LLA using another model, and found that when a young individual is in a relaxed state, similar to the aging spine, the IP level will descend and drive LLA down with it. Hence, the matching relationships between LLA and pelvic morphology and IP level should be comprehensively considered, and surgeons should reconstruct an ideal location of the LLA *via* the individual PI and IP values during ASD correction surgery.

In addition to the proper position of the LLA, a reasonable lumbar shape should include two arcs above and below the apex (ULL and LLL). It has been reported that LLL, geometrically equal to SS, is the most important part that determines the magnitude of global lordosis (7, 10). Herein, no significant relationship was found between LLL and ULL, indicating that the two arcs are independently growing structures; furthermore, the correlation between LLL and LL (*r_s_*_ _= 0.854) is stronger than that between ULL and LL (*r_s_*_ _= 0.594). Notably, it was found that LLL was also modulated by PI and IP (LLL = 0.58 * PI − 0.88 * IP + 18.86, *R*^2 ^= 0.659), which suggests that a low PI and low IP level is associated with a flat LLL. Conversely, individuals with a larger PI and higher IP level will recruit more vertebrae to form a larger LLL and higher LLA to maintain spinopelvic balance in the sagittal plane. This study also found that the ULL, with an average of 20.9°, is not correlated with PI or IP. Our results are consistent with Roussouly et al. (7), who reported that the ULL was relatively constant (average 21.5°) among the different Roussouly types. Besides, TK was significantly positively correlated with ULL (*r_s_* = 0.629, *P* < 0.01, TK = 1.20*ULL + 15.23, *R*^2 ^= 0.426), indicating that ULL is the pedestal of thoracic kyphosis. Hence, the magnitude of TK across individuals is largely determined by the difference in ULL. This needs to be noted during ASD surgery because overcorrected ULL requires a large TK to match with it, which may lead to the occurrence of PJK (18, 21).

**Figure 6:**
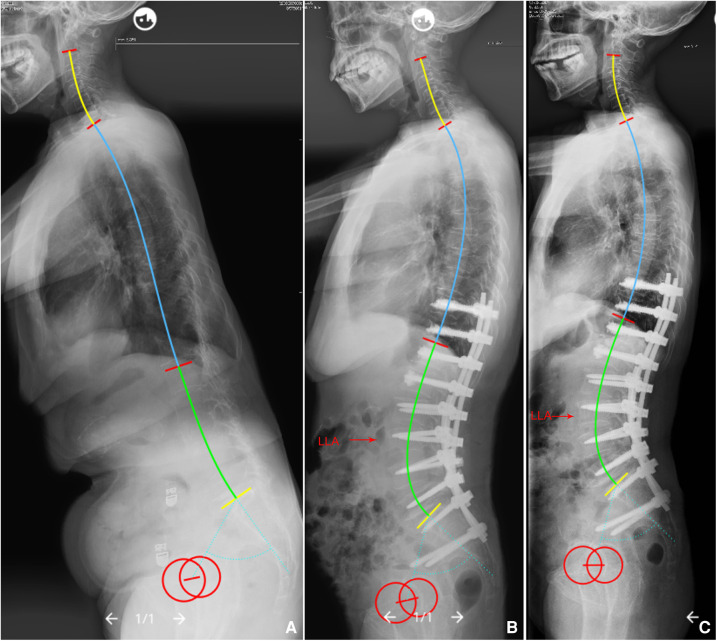
**(A**) A 68-year-old female with global kyphosis, PI = 63.2°. We planned to choose T11/12 as the IP, so that the LLA should be predicted to be L3 (ideal LLA = 0.23*11.5 − 0.03*63.2 + 14.45 = 15.2), ideal LASO = 0.38*63.2 − 2.09*11.5 + 53.62 = 53.6 mm, and the LLL should be corrected to be 45.4° (ideal LLL = 0.58*63.2 − 0.88*11.5 + 18.86 = 45.4°), and the ULL should be corrected to be 20°. (**B**) Immediate postoperative x-rays, actual LLA (L3), LASO (57.4 mm), LLL (46.2°), and ULL (20.1°) were nearly equal to the theoretical values. (**C**) No mechanical complications at the 2-year follow up. PI, pelvic incidence; IP, inflection point; LLA, apex of lumbar lordosis; LASO, the horizontal offset between the plumb line of the lumbar apex and that of the posterosuperior corner of S1; LLL, lower lumbar lordosis; ULL, upper lumbar lordosis.

**Figure 7:**
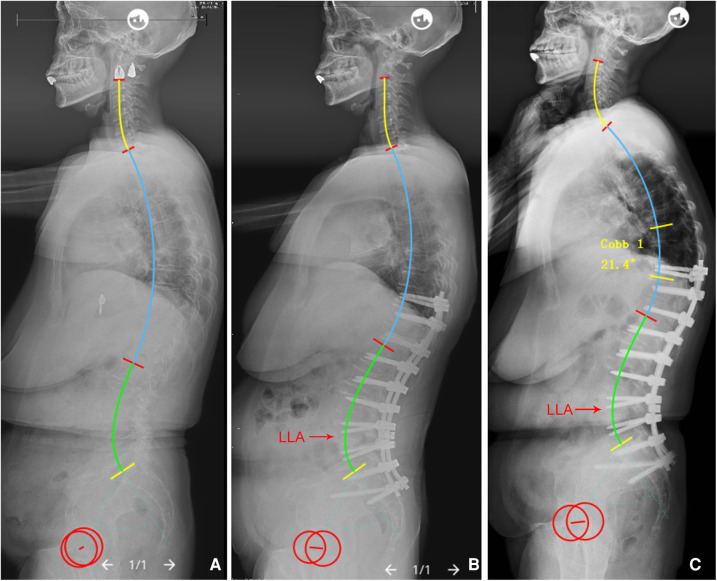
**(A**) A 61-year-old female with adult spinal deformity, PI = 61.3°. (**B**) Immediate postoperative x-rays, IP = 12, the LLA (16 or L4), LASO (43.2 mm), LLL (34.8°), and ULL (32.1°) were not matched to the ideal values (LLA = 0.23*12 − 0.03*61.3 + 14.45 = 15.4, LASO = 0.38*61.3 − 2.09*12 + 53.62 = 51.8 mm, LLL = 0.58*61.3 − 0.88*12 + 18.86 = 43.9°, and ULL = 20°). (**C**) PJK occurred during 6-month follow up. PI, pelvic incidence; IP, inflection point; LLA, apex of lumbar lordosis; LASO, the horizontal offset between the plumb line of the lumbar apex and that of the posterosuperior corner of S1; LLL, lower lumbar lordosis; ULL, upper lumbar lordosis.

Herein, a quantitative method was used to describe the phenomenon that the lumbar shape is regulated by PI and IP based on the Roussouly classification. Results of the comparison of actual LLA, LASO and LLL(SS) with their predicted values revealed no significant differences, indicating that the developed formulae have good predictive effects. It is worth mentioning that the novel formulae can help surgeons better understand the regulatory mechanisms of the individual sagittal lumbar profile in asymptomatic population, and provide a good reference for restoring an optimal sagittal alignment. Namely, ideal LLA, LASO and LLL (SS) can be predicted using these models after designing the IP based on patient's PI (Table 3); then, by simulating the position and magnitude of osteotomy, and the rod contour, surgeons can accurately reconstruct postoperative sagittal lumbar alignment (Figure 6). Moreover, surgeons can identify whether postoperative lumbar alignment is reasonable by assessing whether postoperative lumbar sagittal parameters match the theoretical values derived from PI and IP, and then predict the potential risks of mechanical complications, thereby guiding on follow up and effective intervention (Figure 7).

To the best of our knowledge, this is the first study to calculate with more precision the lumbar apex using both PI and IP simultaneously in a relatively large sample of asymptomatic population. However, the study had some limitations. First, given that this is a single center research and the age of the selected population does not follow a normal distribution, which may result in selective bias, the obtained results may not necessarily be applicable to other regions and clinical contexts. Therefore, multicenter studies should be conducted to confirm our conclusions. Second, subjects included in this study were predominantly young and middle-aged population. According to a previous study (22), the sagittal parameters matching age should be considered in degenerative lumbar fusion surgery. However, it has also been recommended that the goal of sagittal lumbar realignment should refer to the average of sagittal parameters in young adults because the elderly may suffer some loss of lordosis due to weak back muscles after correction surgery (8). Finally, unlike PI, IP is admittedly not completely constant and may have pathological variation before surgery. Therefore, to restore a more reasonable lumbar shape, surgeons should design IP according to its normal range.

## Conclusion

This study has shown that the specific lumbar shape should be modulated by pelvic morphology and IP level. We established predictive formulae for ideal sagittal lumbar profile based on individual PI and IP, with the overarching goal of helping surgeons to better comprehend the regulatory mechanisms of the individual sagittal lumbar alignment, and design a precise and personalized corrective plan.

## Data Availability

The original contributions presented in the study are included in the article/Supplementary Material, further inquiries can be directed to the corresponding author/s.
